# Autism and Children: Diagnosis, Functional Profiles and Intervention

**DOI:** 10.3390/children10030522

**Published:** 2023-03-08

**Authors:** Antonio Narzisi, Yurena Alonso-Esteban, Francisco Alcantud-Marín

**Affiliations:** 1 Department of Child Psychiatry and Psychopharmacology, IRCCS Stella Maris Foundation, 56812 Pisa, Italy; 2 Department of Psychology and Sociology, University of Zaragoza, 44003 Teruel, Spain; 3 Department of Developmental and Educational Psychology, University of Valencia, 46010 Valencia, Spain

In the last forty years, approaches to and the social perception of autism have changed significantly. In the 1970s and 1980s, a prevalence of 4/10,000 was estimated [1]; today, that estimate is between 1 and 2% [2,3]. In the 1970s and well into the 1980s, most autistic people were considered to need lifelong residential or institutional care. Only 2% of those diagnosed would have had the opportunity to achieve independent living [1,4].

In the nineties, autism was still defined as “a severe and chronic developmental disorder, which with current knowledge has no cure, its symptoms manifest themselves in different ways at different ages, accompanying the person during the whole life cycle” (p. 37, [5]). Currently, it is estimated that between 3% and 25% of people initially diagnosed as autistic present competences in social and communicative areas during adulthood similar to those of average development [6,7], and 1% of people diagnosed as autistic before the start of schooling reach higher education [8].

Autism, or autism spectrum disorder (ASD), is nowadays considered one of the most common neurodevelopmental disorders. In the last twenty years, significant research has been undertaken, which has resulted in a very significant increase in the number of publications [9]. The research developed around ASD is undoubtedly multidisciplinary and heterogeneous by nature. That is, heterogeneous in terms of the perspective or vision from which the research originates, but also heterogeneous in terms of the methodology and level of rigor of the results [10].

One of the areas of research where most effort has been made and continues to be made is the etiological determination and pathogenesis of the disorder. It is accepted that ASD has a biological origin with a genetic basis [11], with research focusing on the complex process of human neurodevelopment and the risk factors that may influence it. This Special Issue presents an interesting study on how the immune system may be involved in the “equation” of the etiology of autism [12]. Studies on the etiology provide valuable information on how the genesis of the disorder may occur, but much research is still needed to translate the knowledge gained into clinical practice.

The lack of biological markers for the detection of the disorder means that research focuses on early warning signs in order to initiate early intervention. Posar and Visconti [13] develop a theoretical review of the importance of early signs related to motor development. They hypothesize that motor development and communication are connected, and their dysfunction plays an important role in the pathogenesis of autism. Early motor disturbances in these children can manifest as anything from a delay in motor development to the presence of atypical movements. These alterations can be taken as warning signs and can trigger interventions under the hypothesis that an early normalization of motor development could reduce the symptoms of ASD by acting on common or proximal brain areas. From this perspective, Abdel Ghafar et al. [14] show how autistic children present body instability due to proprioceptive sensory impairment. This work points in the same direction as others [15], in which it is shown that dysfunctions in the motor development of children with ASD, such as a delay in the sitting position or later and dysfunctional crawling, are more present in autistic children. Dysfunctions or motor clumsiness in autistic children are also related to lower communicative skills and social functioning [16], which opens an interesting line of intervention and research.

Existing evidence recognizes the effectiveness of early intervention with techniques and methods based on the application of the principles of behavior analysis and child development [17,18,19], as well as NDBI (naturalistic developmental behavioral interventions) programs with an emphasis on the use of typical developmental principles, structured learning scenarios, stimulus control, development of routines, and natural environments, among others [20].

Play is a substantial element of intervention, particularly with young children [21]. The general trend in early intervention is toward intervention in the precursors of communication and social interaction and language. There is some belief that bilingualism overloads language development in autistic children [22], although evidence points in the opposite direction [23]. In this Special Issue, Hashim et al. [24] study the language learning styles (in particular, English language learning) of autistic children. Giangiacomo et al. [25] present a pilot study in which they attempt to demonstrate the usefulness of a neuro-psychomotor approach in the early years of life in relation to the development of theory of mind (ToM) and emotion recognition. The development of joint attention as a precursor to the development of communication and language or ToM has attracted the attention of numerous researchers. In this Special Issue, Montagut-Asunción et al. [26] present a longitudinal study showing how the development of joint attention plays a fundamental role in defining the manifestations of ASD.

Results of effective interventions based on psycho-educational models have been reported [11,27]. However, studies on the implementation of evidence-based practices and the training of professionals show that there is a long way to go [28].

The interest in the specific study of the development of certain skills in autistic children and adolescents aims to provide greater knowledge about the disorder and its trajectory throughout life and, in turn, to improve intervention. The individual profiles of autistic people are very heterogeneous, including delays in the onset of language, its use, sensory alterations, the presence of concurrent intellectual disability, etc. [29]. This heterogeneity has multiple consequences for everyday life and for the development of knowledge about the disorder. Undoubtedly, the development of communicative function and language is one of the most important areas of study and intervention due to the wide range of consequences for functioning in daily life [30], social relationships [31], and overall mental health and quality of life [9,32]. Ong et al. [33] provide evidence of the benefits of parental involvement in children’s friendship training (CFT) for the development of social, communication and friendship skills. Parental involvement in intervention programs is becoming more and more intense. It was first justified by economic issues, and then by enhancing skills in natural spaces. Multiple instruments have been developed to measure the perceived severity of symptoms (Autism Impact Measurement [34] and Childhood Autism Ratings Scale [35], among others). Lira-Rodriguez et al. [36] demonstrate that parental overload does not depend exclusively on the severity of symptoms, so that an integrated parent–child intervention is advisable. In intervention programs aimed at fostering communication, language and social skills, the mediating role of parents is increasingly valued. A critical issue has been the use of parents as informants of their child’s symptoms. Evidence on the reliability of parents’ assessment of ASD symptom severity has already been described [37]. In this issue, Jagadeesan et al. [38] provide a study with a sample of 9573 children where they demonstrate a consistent relationship between parental assessments and the results of questionnaires such as the ATEC (Autism Treatment Evaluation Checklist) or the MSEC (Mental Synthesis Evaluation Checklist).

On the other hand, technological development directly affects interventions with autistic children. The number of applications developed as assistive technology are numerous [39]; however, the therapeutic results are sometimes questionable. Among the suggested advantages is the increased motivation generated by screens. Evidence suggests that using multimedia learning models [40] that combine different sensory channels can benefit the learner. In the case of autistic children, these applications should be designed so that they can be personalized. The results provided in the review in this Special Issue by Maseri et al. [41] suggest that some of the apps developed to improve verbal communication in children with ASD are effective. Some are designed as a complement to the intervention of the professional and with the professional, and others are designed to be used by children with parental supervision. In any case, they should be tools to be considered in the framework of a personalized intervention program.

General aspects of daily life and health care should not be left out of research. Aspects related to diet in autistic children are being systematically reviewed for the practical implications of extended research with very uneven and methodologically questionable results. Other preventive health aspects have also been studied, such as oral health. In this issue, Carti et al. [42] report data on the results of a preventive program with personalized digestive support with which they managed to increase collaboration and awareness of the importance of dental hygiene, as well as day hospital settings in relation to routine health care [43].

The large volume of research and the diversity of areas, as the content of this Special Issue demonstrates, makes it necessary to orchestrate some basic principles that allow us to select papers both according to their quality and their specific area of development. The enormous diversity of symptoms and severity levels of autism spectrum disorders makes it necessary to build solid methodological guidelines incorporating not only behavioral measures but also biological measures in order to reach consistent knowledge in a multidisciplinary area and with an object of study that, as its name indicates, is spectral [10].

A recent paper has been published that should give us pause for thought [44]. In clinical studies, intervention generally relies on pre-diagnosis to identify the neurodevelopmental disorder the child has. The design, recruitment of participants and selection of measures and theoretical models are based on the assumption of a homogeneity derived from meeting diagnostic criteria. However, the literature reflects evidence of the heterogeneity of ASD symptoms, and perhaps the over-reliance on sometimes imprecise diagnostic criteria impedes progress in identifying the barriers that children encounter and understanding the underlying mechanisms. Atle et al. [44] question the hegemonic role with which current diagnostic taxonomies of a discreet nature are used in research methodology. The concept of neurodiversity [45] involves accepting the fact that human beings vary in neurological composition and structuring and that this variability dictates to some extent how we process information and how we store it. Children with the same diagnostic label, autism, can vary widely in the extent, nature, and impact of their symptoms. At present, an alternative or complementary transdiagnostic approach could be developed that is characterized by defining disorders in terms of dimensions rather than discrete categories [44]. It is these dimensions that could direct intervention, rather than the classical diagnostic label that does little to help.

## Data Availability

Not applicable.

## References

[1] DeMyer M.K., Hingtgen J.N., Jackson R.K. (1981). Infantile autism reviewed: A decade of research. Schizophr. Bull..

[2] Autism Europe (2019). People with Autism Spectrum Disorders.

[3] Baio J., Wiggins L., Chistensen D.L., Maenner M.J., Daniels J., Warren Z., Kurzius-Spencer M., Zahorodny W., Robinson-Rosenberg C., White T. (2018). Prevalence of Autism Spectrum Disorder Among Children Aged 8 Years—Autism and Developmental Disabilities Monitoring Network, 11 Sites, United States, 2014. MMWR Morb. Mortal. Wkly. Rep. Surveill. Summ..

[4] Rutter M. (1970). Autistic children: Infancy to adulthood. Semin. Psychiatry.

[5] Frith U. (1991). Autism and Asperguer Syndrome.

[6] Fein D., Barton M., Eigsti I., Kelley E., Naigles L., Schultz R.T., Stevens M., Helt M., Orinstein A., Rosenthal T.E. (2013). Optimal outcome in individuals with a hisotry of autism. J. Child Psychol. Psychiatry.

[7] Helt M., Kelley E., Kinsbourne M., Pandey J., Boorstein H., Herbert M., Fein D. (2008). Can Children with autism recover? Is so, how?. Neuropsychol. Rev..

[8] Newman L., Wagner M., Knokey A.M., Marder C., Nagle K., Shaver D., Wei X. (2011). The Post-High School Outcomes of Young Adults With Disabilities up to 8 Years after High School.

[9] Alcantud-Marín F., Alonso-Esteban Y. (2022). Autism Spectrum Disorders: Bases for Psychoeducational Intervention.

[10] Narzisi A., Alonso-Esteban Y., Masi G., Alcantud-Marín F. (2022). Research-Based Intervention (RBI) for Autism Spectrum Disorder: Looking beyond Traditional Models and Outcome Measures for Clinical Trials. Children.

[11] Filipek P.A., Steinberg-Epstein R., Book T.M. (2006). Intervention for autistic spectrum disorders. NeuroRX.

[12] Alhosaini K., Ansari M.A., Nadeem A., Attia S.M., Bakheet S.A., Al-Ayadhi L., Mahmood H.M., Al-Mazroua H.A., Ahmad S.F. (2021). Dysregulation of Ki-67 Expression in T Cells of Children with. Chidren.

[13] Posar A., Visconti P. (2022). Early Motor Signs in Autism Spectrum Disorder. Children.

[14] Abdel Ghafar M.A., Abdelraouf O.R., Abdelgalil A.A., Seyam M.K., Radwan R.E., El-Bagalaty A.E. (2022). Quantitative Assessment of Sensory Integration and Balance in Children with AutismSpectrumDisorders: Cross-Sectional Study. Children.

[15] Lavenne-Collot N., Jallot N., Maguet J., Degrez C., Botbol M., Grandgeorge M. (2021). Early Motor Skills in Children With Autism Spectrum Disorders Are Marked by Less Frequent Hand and Knees Crawling. Percept. Mot. Ski..

[16] Craig F., Crippa A., Ruggiero M., Rizzato V., Russo L., Fanizza I., Trabacca A. (2021). Characterization of Autism Spectrum Disorder (ASD) subtypes based on the relationship between motor skills and social communication abilities. Hum. Mov. Sci..

[17] Makrygianni M.K., Reed P. (2010). A meta-analytic review of the effectiveness of behavioural early intervention programs for children with autistic spectrum disorders. Res. Autism Spectr. Disord..

[18] Reichow B. (2012). Overview of meta-analyses on early intensive behavioral intervention for young children with autism spectrum disorders. J. Autism Dev. Disord..

[19] Smith T., Iadarola S. (2015). Evidence Base Update for Autism Spectrum Disorder. J. Clin. Child Adolesc. Psychol..

[20] Lang R., Hancock T.B., Singh N.N. (2016). Overview of early intensive behavioral intervention for children with autism. Early Intervention For Young Children With Autism Spectrum Disorder.

[21] López-Nieto L., Compañ-Gabucio L., Torres-Collado L., Garcia-de la Hera M. (2022). Scoping Review on Play-Based Interventions in Autism Spectrum Disorder. Children.

[22] Hampton S., Rabaglliati H., Sorace A., Fletcher-Watson S. (2017). Autism and Bilingualism: A Qualitative Interview Study of Parents’ Perspectives and Experiences. J. Speech Lang. Hear. Res..

[23] Lund E.M., Kohlmeier T.L., Durán L.K. (2017). Comparative Language Development in Bilingual and Monolingual Children with Autism Spectrum Disorder: A Systematic Review. J. Early Interv..

[24] Hashim H., Yunus M., Norman H. (2022). Autism Children and English Vocabulary Learning: A Qualitative Inquiry of the Challenges They Face in Their English Vocabulary Learning Journey. Children.

[25] Giangiacomo E., Visaggi M.C., Aceti F., Giacchetti N., Martucci M., Giovannone F., Valente D., Galeoto G., Tofani M., Sogos C. (2022). Early Neuro-Psychomotor Therapy Intervention for Theory of Mind and Emotion Recognition in Neurodevelopmental Disorders: A Pilot Study. Children.

[26] Montagut-Asunción M., Crespo-Martin S., Pastor-Cerezuela G., D’Ocon-Gimenez A. (2022). Joint Attention and Its Relationship with Autism Risk Markers. Children.

[27] Howlin P., Fleischhacker W., Brooks D. (2005). The effectiveness of interventions for children with autism. Neurodevelopmental Disorders.

[28] Larraceleta A., Castejon L., Iglesias-García M.T., Núñez J.C. (2022). Assessment of Public Special Education Teachers Training Needs on Evidence-Based Practice for Students with Autism Needs on Evidence-Based Practice for Students with Autism. Children.

[29] Lord C., Brugha T.S., Charman T., Cusack J., Dumas G., Frazier T., Jones E.J.H., Pickles A., State M.W., Taylor J.L. (2020). Autism spectrum disorders. Nat. Rev. Dis. Prim..

[30] Duncan A., Ruble L.A., Meinzen-Derr J., Thomas C., Stark L.J. (2018). Preliminary efficacy of a daily living skills intervention for adolescents with high-functioning autism spectrum disorder. Autism.

[31] Elmose M. (2020). Understanding loneliness and social relationships in autism: The reflections of autistic adults. Nord. Psychol..

[32] Dijkhuis R.R., Ziermans T.B., Van Rijn S., Staal W.G., Swaab H. (2016). Self-regulation and quality of life in high-functioning young adults with autism. Autism.

[33] Ong S.Y., Roslan S., Ahmad N.A., Ayub A.F.M., Chen L.P., Taresh S.M. (2022). Parents’ Experience in Children’s Friendship Training Programme for Their Children with Autism Spectrum Disorder: A Qual itative Inquiry. Children.

[34] Kanne S.M., Mazurek M.O., Sikora D., Bellando J., Branum-Martin L., Handen B., Katz T., Freedman B., Powell M.P., Warren Z. (2014). The Autism Impact Measure (AIM): Initial development of a New Tool for treatment outcome measuremente. J. Autism Dev. Disord..

[35] Schopler E., Van Bourgondien M., Wellman G., Love S. (2010). Chilldhood Autism Rating Scale-Seconf Edition (CARS-2): Manual.

[36] Lira Rodríguez E., Pascual R., Sanclemente M., Martín-Hernández P., Gil-Lacruz M., Gil-Lacruz A. (2022). The Influence of ASD Severity on Parental Overload: The Moderating Role of ParentalWell-Being and the ASD Pragmatic Level. Children.

[37] Miller L.E., Perkins K.A., Dai Y.G., Fein D.A. (2017). Comparison of parent report and direct assessment of child skills in toddlers. Res. Autism Spectr. Disord..

[38] Jagadeesan P., Kabbani A., Vyshedskiy A. (2022). Parent-Reported Assessment Scores Reflect the ASD Severity Level in 2- to 7-Year-Old Children. Children.

[39] Alcantud-Marín F., Alonso-Esteban Y., Alcantud-Marin F. (2013). Technological tools in psychoeducational intervention for children with autism spectrum disorders. Autism Spectrum Disorders: Detection, Diagnosis and Early Intervention.

[40] Mayer R.E., Mayer R.E. (2014). Cognitive Theory of Multimedia Learning. Cambrisge Handbook of Multimedia Learning.

[41] Maseri M., Mamat M., Yew H.T., Chekima A. (2022). The Implementation of Application Software to Improve Verbal Communication in Children with Autism Spectrum Disorder: A Review. Children.

[42] Carti E., Pasini M., Pardossi F., Capotosi I., Narzisi A., Lardani L. (2022). Oral Health Preventive Program in Patients with Autism Spectrum Disorder. Children.

[43] Davico C., Marcotulli D., Succi E., Canavese C., Bodea A., Pellegrino M., Cuffari E., Cudia V., Svevi B., Amianto F. (2023). Working with Children with Autism Undergoing Health-Care Assessments in a Day Hospital Setting: A Perspective from the Health-Care Professionals. Children.

[44] Atle D.E., Holmes J., Kievit R., Gathercole S.E. (2022). Annual Research Review: The transdiagnostic revolution in neurodevelopmental disorders. J. Child Psychol. Psychiatry.

[45] Milton D., Ribout S., Murray D., Martin N., Mills R. (2020). The Neurodiversity Reader: Exploring Concepts, Lived Experiences and Implications for Practice.

